# Food delivery services for enhancing participation in the Special Supplemental Nutrition Program for Women, Infants, and Children (WIC) in the United States: bridging the WIC-eligible access gap

**DOI:** 10.3389/fpubh.2026.1756682

**Published:** 2026-03-27

**Authors:** Jingjing Gao, Muinat Abolore Idris, Lilian O. Ademu, Bryan Colby Griffin, Yue Zhang, Yun Hang, Gabriela A. Gallegos

**Affiliations:** 1 Department of Management, Policy & Community Health, University of Texas Health Science Center at Houston School of Public Health, Houston, TX, United States; 2 Department of Health Promotion and Behavioral Sciences, School of Public Health, University of Texas Health Science Center at Houston, Houston, TX, United States; 3 Texas A&M AgriLife Research Center at El Paso, TX, United States; 4 Department of Environmental and Occupational Health Sciences, School of Public Health, University of Texas Health Science Center at Houston, Houston, TX, United States; 5 Department of Biostatistics and Data Science, School of Public Health, University of Texas Health Science Center at Houston, Houston, TX, United States

**Keywords:** diet, food delivery, health policy, public policy, WIC

## Abstract

**Background:**

Diet-related health disparities among U.S. children persist despite the availability of the Special Supplemental Nutrition Program for Women, Infants, and Children (WIC) benefits to eligible women, infants, and children. Participation remains suboptimal: only 51.2% of eligible individuals were enrolled in 2021. Transportation and food-environment constraints pose salient barriers, especially for caregivers of infants in food deserts. Emerging online ordering and delivery options may reduce access frictions, but grocery delivery coverage is limited, and fees are often not covered by benefits.

**Methods:**

We linked USDA Food and Nutrition Service state-level WIC participation data (November 2024 vs. September 2025) with demographic, economic, and political indicators to examine whether food retail delivery availability is associated with changes in WIC participation and whether this association varies by poverty and unemployment context. Delivery availability was coded as a binary indicator based on state announcements of online ordering/delivery pilots. Descriptive statistics characterized cross-state variation. Ordinary least squares models with robust standard errors were used to test associations, including an interaction between delivery availability and the poverty and unemployment rates; marginal predictions visualized the interaction.

**Results:**

Only 2 of 50 states (4%) had active WIC online ordering/delivery options during the study window. The average percentage change in participation rate was modest (mean = 1.23; SD = 2.70). In the baseline model, the poverty rate was positively associated with the change in participation (*β* = 0.62, *p* < 0.05). Adding the interaction improved model fit (R^2^ = 0.47) and revealed a significant positive moderation: states with delivery experienced larger gains in participation as poverty increased (Delivery × Poverty *β* = 11.83, *p* < 0.001) or as the unemployment rate increased (Delivery × Unemployment β = 6.56, *p* < 0.001). Other covariates were not statistically significant. Margins plots showed that at higher poverty levels (>15%), predicted participation gains in delivery states exceeded those in non-delivery states by more than 75 percentage points. Margins plots also indicated that when unemployment rates rise above 5%, the expected increase in participation in delivery states is more than 10 percentage points higher than in non-delivery states.

**Conclusion:**

WIC-eligible food delivery availability is associated with greater increases in participation in higher-poverty states or higher-unemployment states, suggesting that modernization can mitigate structural access barriers for families with young children. Policy actions, such as subsidizing delivery fees, expanding retailer participation, and providing digital access supports, could enhance equitable reach. Future work should leverage longitudinal and individual-level data to test mechanisms, evaluate fee-subsidy pilots, and assess heterogeneity by rurality, race/ethnicity, and family structure.

## Introduction

Diet-related conditions among U.S. children remain a persistent public-health concern that is closely tied to structural inequities in access to nutritious foods (1–4). The Special Supplemental Nutrition Program for Women, Infants, and Children (WIC) is designed to mitigate these inequities by providing targeted food benefits, nutrition education, and breastfeeding support to low-income pregnant and postpartum women, infants, and young children. WIC is a federally funded nutrition assistance program in the United States designed to support the health of low-income pregnant women, postpartum women, infants, and children under age five who are at nutritional risk. The program provides monthly food benefits for specific nutritious foods, nutrition education, breastfeeding support, and referrals to health and social services. WIC has been widely recognized as one of the most effective maternal and child health programs in the United States, with evidence linking participation to improved birth outcomes, better childhood nutrition, and reduced food insecurity. Previous studies show that participation in WIC is associated with improved birth outcomes, healthier dietary patterns, and greater breastfeeding initiation and duration (5–10). Yet substantial gaps in reach persist: in 2021, only 51.2% of eligible individuals, approximately 12.13 million people, were enrolled (11). Structural barriers-including transportation challenges, time constraints for caregivers with young children, and limited access to authorized retailers-continue to hinder program enrollment and consistent benefit use. These access challenges have prompted increasing interest in digital modernization strategies, such as online ordering and home delivery of WIC-approved foods, which may reduce logistical barriers and improve program reach among underserved populations.

A growing body of research points to transportation and the difficulties in bringing the infant or young child to the store as key barriers to enrolling in and consistently using WIC benefits (12–18). Families living in food deserts, areas with limited geographic access to affordable, healthy food, face longer travel times, higher indirect costs, and constrained retailer choice (19–22). Transportation challenges are compounded for households without reliable vehicle access or adequate public transit and can contribute to poorer dietary quality and higher risk of obesity and diet-related disease among children (16, 23–25). These constraints are particularly salient for single mothers with young children, who may face substantial financial burdens from routine grocery trips.

In response, online ordering and home delivery have emerged as promising strategies to reduce access frictions for nutrition-assistance programs. Early evidence suggests that delivery can mitigate transportation barriers, improve convenience, and enhance adherence to program dietary guidance (26–28). However, implementation challenges remain. Programs operate within tight budgets, and delivery/service fees are often not covered by benefits, which can undermine the overall purchasing power of WIC benefits for low-income families (29). Moreover, digital literacy and device/connectivity constraints may limit equitable uptake, underscoring the need for participant and staff training to ensure inclusive access (30–32).

Against this backdrop, this study uses recent state-level data on WIC participation to examine whether access to online ordering/delivery is associated with changes in participation, and whether the association varies by poverty and unemployment context. By linking publicly available WIC participation data with state socioeconomic and political characteristics, we assess whether delivery availability moderates the relationship between state poverty rates and year-over-year changes in WIC participation. Findings can inform ongoing modernization efforts to reduce structural barriers, extend equitable program reach, and improve health outcomes for WIC participants.

## Methods

### Data WIC participation

Participation data for this analysis were obtained from the U.S. Department of Agriculture (USDA) Food and Nutrition Service (FNS) public database on the WIC website, https://www.fns.usda.gov/pd/wic-program. The dataset titled *“WIC Program: Total Participation”* provides state- and territory-level monthly enrollment statistics for the WIC program. The dataset used in this study reflects data as of March 4th, 2026, and includes participation counts for November 2024 and September 2025, along with calculated percent changes between these two time points (November 2024 vs. September 2025) and year-over-year changes (May 2025 vs. May 2024) for visualization. These administrative data are compiled and released monthly by FNS based on reports submitted by state WIC agencies. The dataset provides a comprehensive and standardized measure of WIC participation across all U.S. states and territories, supporting national and subnational trend analyses in maternal and child nutrition program engagement.

### WIC delivery service availability

To capture state-level differences in WIC modernization, we created a binary indicator for WIC delivery service availability based on publicly available information from the FNS and state WIC program websites. Specifically, we reviewed official announcements, news releases, and government communications describing the implementation of online ordering and delivery options for WIC participants through authorized retailers such as Walmart. Delivery availability was coded based on official program announcements and pilot implementation, not outreach intensity. The study period was selected to capture participation trends immediately before and after the introduction of online ordering and delivery pilot programs in Massachusetts and Washington, which began in April 2025. Using administrative data spanning November 2024 through September 2025 allows the analysis to examine early changes in WIC participation associated with the initial implementation of these modernization efforts.

Each state or territory was assigned a value of 1 if it had implemented or was piloting an online ordering program for WIC-approved foods that explicitly included delivery or pickup options (e.g., Massachusetts and Washington). States without an active or announced online-ordering/delivery program were assigned a value of 0. This binary variable-Delivery Service-thus represents whether WIC participants in each state had access to a verified online ordering and delivery mechanism (1 = available, 0 = not available) based on official FNS and state public disclosures (see Table 1).

**Table 1:** WIC delivery service availability.

State	Launch date	Details
Massachusetts	April 1, 2025 (Massachusetts Department of Public Health)	The state announced that WIC participants could now use their WIC card to order WIC-approved foods online at Walmart (for pickup or delivery) at ~48 Walmart stores statewide (Fall River Reporter: https://fallriverreporter.com/massachusetts-health-officials-launch-first-ever-online-grocery-ordering-program-for-wic-participants-including-walmart/?utm_source=chatgpt.com).
Washington	April 1, 2025 (Washington State Department of Health)	The state launched a pilot in partnership with Walmart (67 stores) allowing WIC participants to order for pickup, delivery, or shipping (Washington State Department of Health: https://doh.wa.gov/newsroom/washington-wic-chosen-online-ordering-pilot-program-walmart?utm_source=chatgpt.com).

### State-level demographic and economic indicators

State-level demographic and socioeconomic data (2023) were compiled from publicly available sources to contextualize policy adoption. Population size, density, educational attainment, race and ethnicity, median income, and poverty rates were obtained from the U.S. Census Bureau’s American Community Survey. Urban–rural distribution data also came from the Census Bureau. Economic indicators, including GDP per capita and unemployment rates, were sourced from the U.S. Bureau of Economic Analysis and Bureau of Labor Statistics. Together, these data provide a comprehensive overview of state-level socioeconomic contexts relevant to delivery service policy analysis.

### Statistical methods

We first conducted descriptive analyses to summarize the distribution of all state-level variables. Means, standard deviations, and observed ranges were calculated for continuous measures, including percent changes in WIC participation, poverty rate, unemployment rate, GDP per capita, racial/ethnic composition, urbanization, and educational attainment. Categorical variables, such as delivery service availability and state political party majority, were described using frequency and percentage distributions. These descriptive statistics provided an overview of socioeconomic and political contexts across the 50 U.S. states before regression modeling.

Ordinary least squares (OLS) regression was used because the dependent variable, percent change in WIC participation between November 2024 and September 2025, is a continuous outcome that met the assumptions of linearity and independence. Multicollinearity diagnostics indicated acceptable variable independence (all variance inflation factors < 5), supporting model stability. The Breusch-Pagan/Cook-Weisberg test for heteroskedasticity rejected the null hypothesis of constant variance (*χ^2^* (1) = 9.70, *p* = 0.0018), suggesting unequal residual variance across fitted values. To account for this, all models were estimated using robust standard errors, which provide consistent estimates of parameter uncertainty under heteroskedasticity. Given that all other OLS assumptions were satisfied, the use of OLS with robust standard errors is appropriate for this cross-sectional state-level analysis (33).

To examine whether the association between state poverty rates and changes in WIC participation differed by delivery-service availability, we estimated an interaction model using OLS regression. The dependent variable was the percent change in WIC participation from November 2024 to September 2025, as reported by the FNS. The main independent variable was delivery availability (coded 1 = state had an active or pilot online ordering/delivery program for WIC participants as of August 2025; 0 = no program). An interaction term between delivery availability and state poverty rate was included (*i.delivery ## c.poverty_rate* in Stata). Predicted values and 95% confidence intervals were computed using the margins and marginsplot commands in Stata 18, holding all other covariates at their sample means. The margins plot displays the predicted percent change in WIC participation across poverty rates (5–25%) separately for states with and without WIC delivery services. Additionally, an interaction term between delivery availability and state unemployment rate was also included (*i.delivery ## c.unemployment_rate* in Stata) to examine whether the association between state unemployment rates and changes in WIC participation differed by delivery-service availability.

### Software

Data cleaning and management were conducted in R (version 4.5.1). A spatial visualization map was created in ArcGIS Pro (version 3.2) (34). All descriptive statistics, econometric modeling, and diagnostics were performed using Stata (version 18.5).

## Results

Table 2 presents the descriptive statistics for all state-level variables (N = 50). Between November 2024 and September 2025, the mean percent change in WIC participation was modest (mean = 1.23, SD = 2.70), ranging from a 0.54% decline to a 10.68% increase across states. GDP per capita averaged $55,756, with substantial variation across states ($42,000–$72,000). States were predominantly White (mean = 74.5%), with average Hispanic and Black population shares of approximately 11% each. On average, 72% of residents lived in urban areas, and 31% held a college degree. Regarding political context, 34% of states were under Democratic control, 8% had divided governance, and 58% were Republican-controlled. The mean state poverty rate was 13.2% (range: 8.8–20.0%), and the average unemployment rate was 4.2%. Only two states (4%) had implemented online ordering or delivery services for WIC participants as of March 2026.

**Table 2:** Descriptive statistics of state-level variables (*N* = 50).

Variable	*n* (%)	Mean	SD	Min	Max
Percent change in WIC participation (November 2024 vs. September 2025)		1.23	2.70	−0.54	10.68
Delivery service available					
Yes	2 (4)	2			
No	48 (96)				
Poverty rate (%)		13.20	2.66	8.8	20.0
Unemployment rate (%)		4.21	0.83	2.8	6.5
GDP per capita (USD)		55,756	9,053.9	42,000	72,000
Hispanic population (%)		10.87	9.84	1.2	48.8
Black population (%)		10.87	9.50	0.3	37.6
White population (%)		74.52	11.65	48.0	93.8
Urban population (%)		72.32	16.17	38.9	95.0
College degree (%)		31.24	5.74	20.7	43.5
Political Party control (1 = Democratic, 1.5 = Divided, 2 = Republican)		1.62	0.47	1	2
Democratic	17 (34%)				
Divided	14 (8%)				
Republican	29 (58%)				

The data represent 50 U.S. states. Delivery availability indicates whether states had implemented online ordering or delivery programs for WIC participants as of August 2025 (4% = Yes).

**Figure 1:**
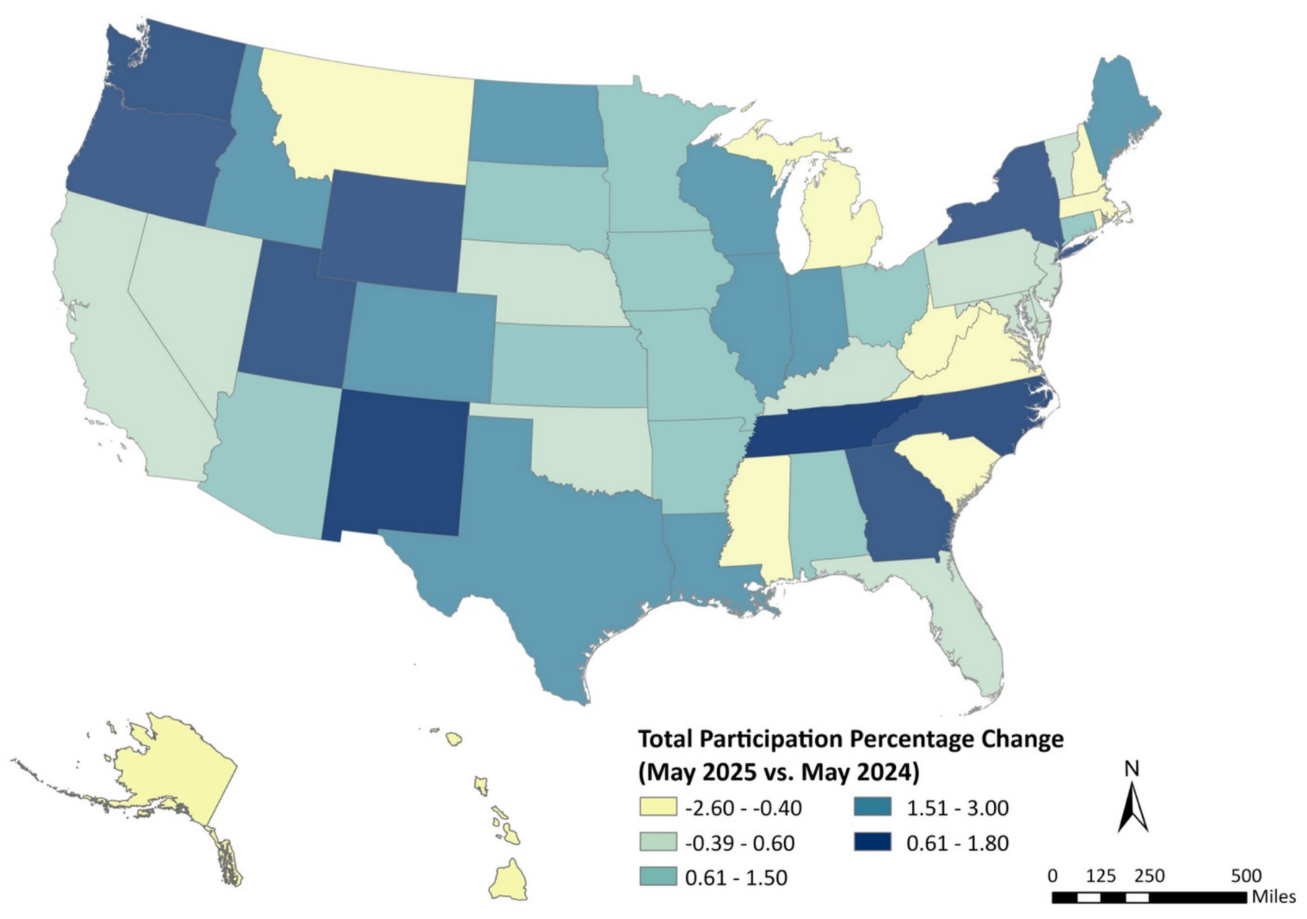
Year-to-year percent change in WIC total participation, May 2025 vs. May 2024, by U.S. state.

Between May 2024 and May 2025, WIC participation trends varied considerably across the United States (Figure 1). Several western and mid-Atlantic states, including Washington, Oregon, Colorado, New Mexico, and Virginia, showed the largest gains, with total participation increasing by 1.5 to 3.0%. Moderate growth (0.6 to 1.5%) was observed across much of the central U.S. and portions of the Southeast. In contrast, participation declined in a subset of states, including Alaska, Tennessee, and parts of New England, where total enrollment decreased by up to 2.6%. These spatial differences suggest uneven recovery and engagement patterns across states, potentially influenced by differences in outreach strategies, digital service expansion (such as online benefit redemption), and local socioeconomic conditions.

**Figure 2:**
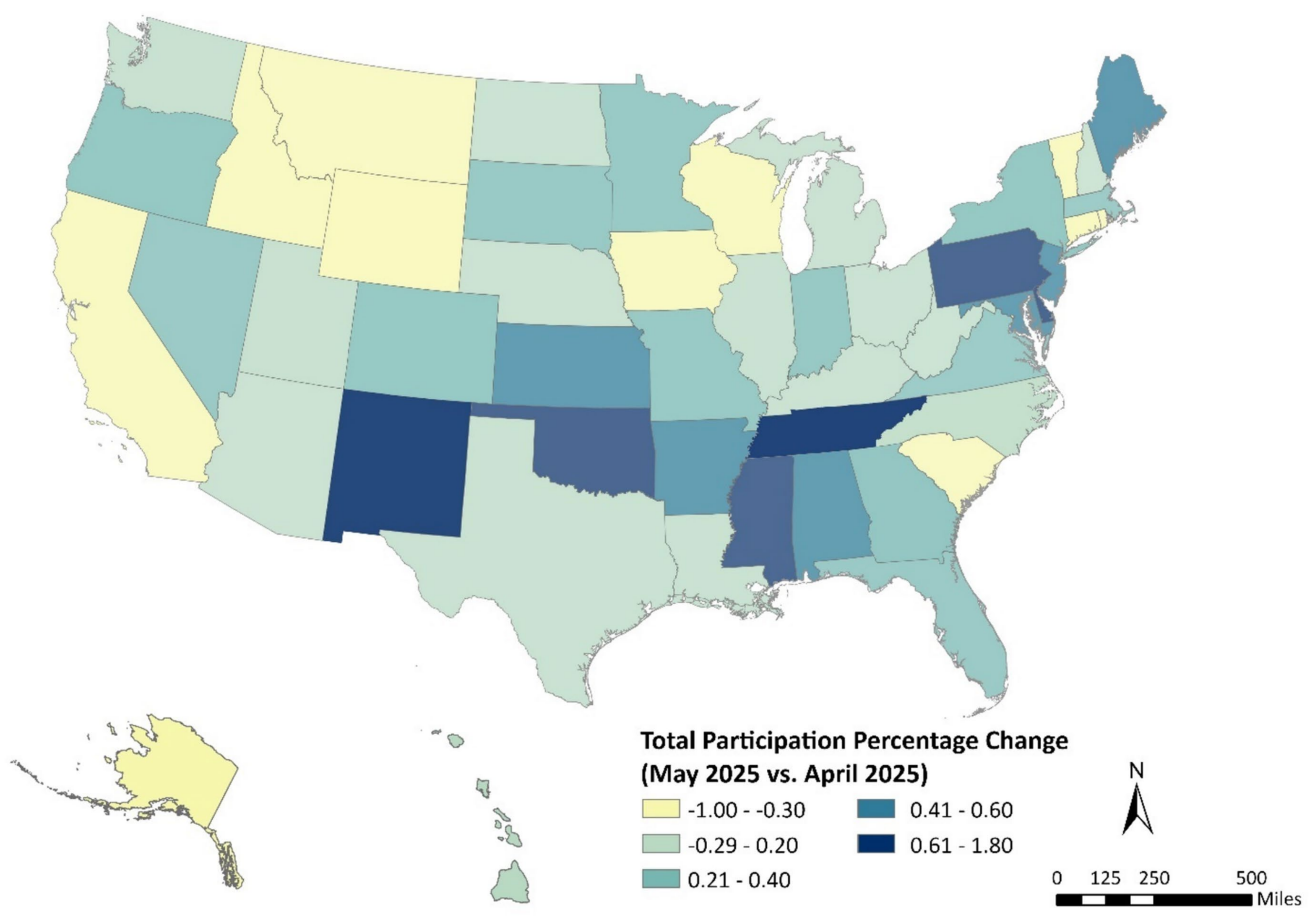
Short-term change in WIC participation rates across U.S. states, April–May 2025.

Month-to-month changes in WIC participation between April and May 2025 show a modest but geographically varied pattern across the United States (Figure 2). Most states recorded small increases of 0.2–0.6%, while the largest gains were concentrated in the South and Mountain West, particularly Tennessee, Oklahoma, and New Mexico, where participation rose by up to 1.8%. Notably, Massachusetts and Washington, both of which recently launched online WIC ordering pilots in partnership with Walmart, also experienced slight participation growth during this period. In contrast, a few states, including Alaska and parts of the Upper Midwest and New England, experienced minor declines (−1.0% to −0.3%).

When compared with the year-over-year trends from May 2024 to May 2025 (Figure 1), the month-to-month map reflects short-term fluctuations rather than broad structural shifts. The annual map showed sustained increases in participation across western and mid-Atlantic states, whereas the May 2025 snapshot highlights localized surges that may coincide with recent program innovations, outreach campaigns, or benefit renewals. Taken together, the two figures suggest that while WIC participation continues to rise nationally, short-term dynamics remain influenced by state-level implementation efforts, particularly in states expanding digital access to benefits such as Massachusetts and Washington.

**Figure 3:**
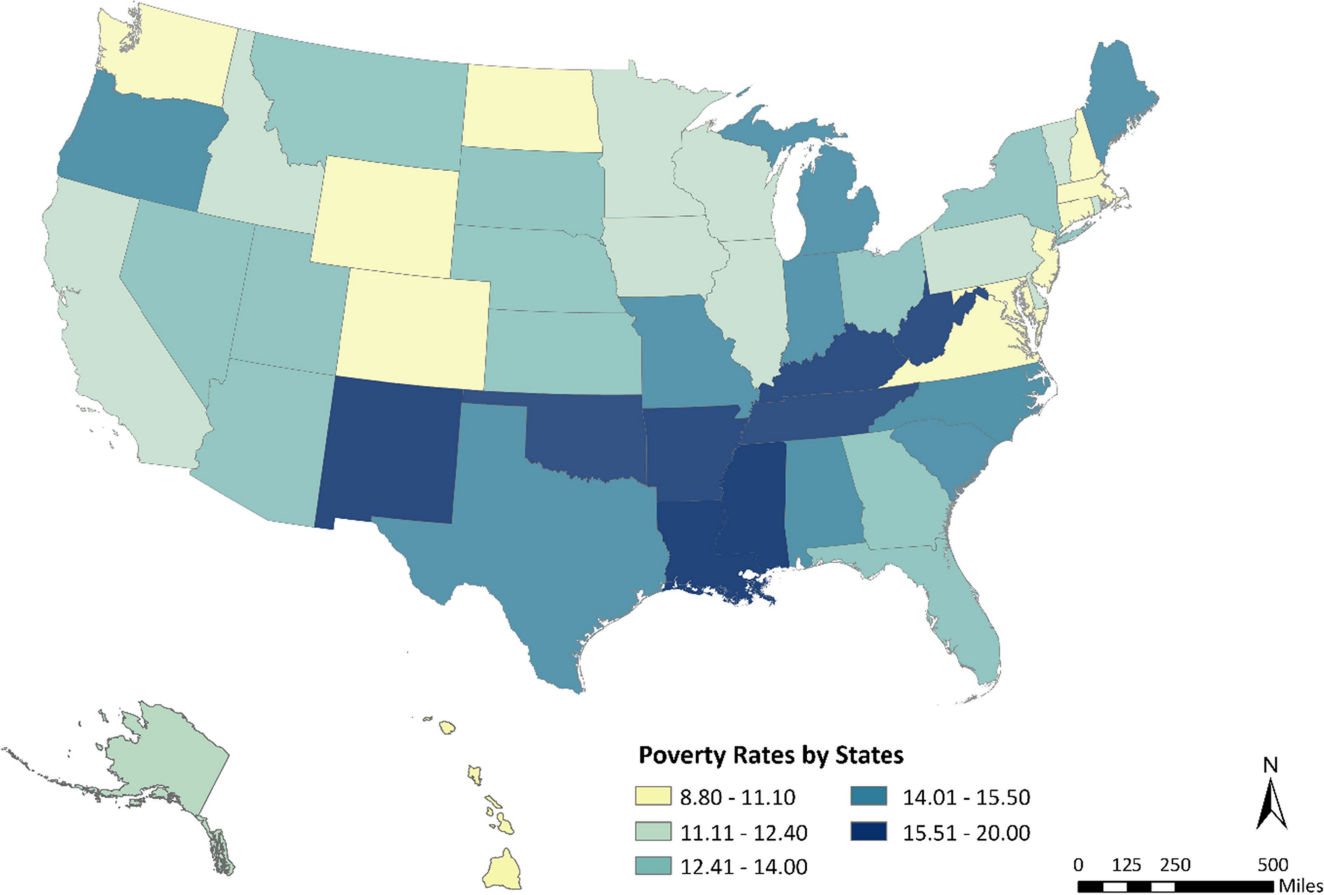
State-level poverty rates in the United States.

The choropleth map (Figure 3) illustrates the geographic distribution of state-level poverty rates across the United States. Darker shades indicate higher poverty levels. Consistent with longstanding regional disparities, the map shows that poverty is most concentrated in the South, particularly in Mississippi, Louisiana, Arkansas, and Alabama, while many states in the Northeast and upper Midwest exhibit substantially lower rates of poverty. Western states display mixed patterns, with relatively low poverty in Utah and Washington but elevated rates in Arizona and New Mexico.

**Figure 4:**
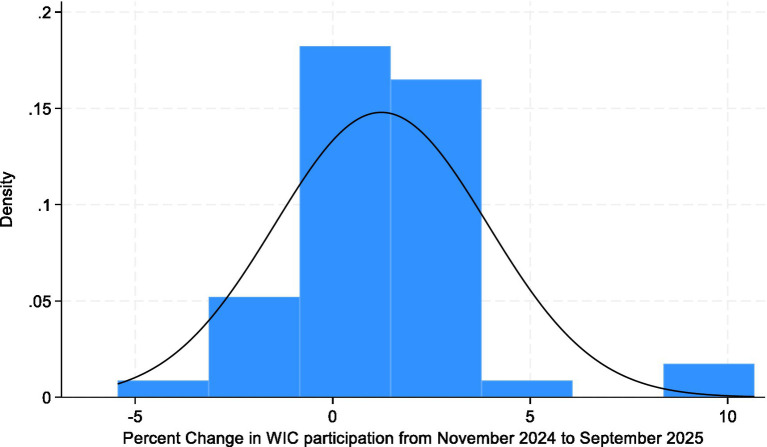
Histogram of percent change in WIC participation across U.S. states, November 2024–September 2025. The histogram illustrates the distribution of the percent change in WIC participation across 50 U.S. states during the study period, with a normal density curve overlaid for comparison.

**Figure 5:**
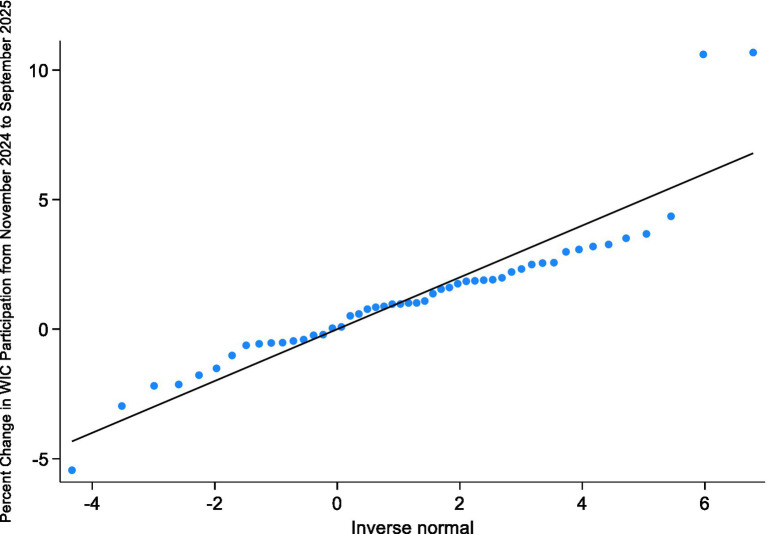
Normal Q-Q plot of percent change in WIC participation across U.S. states. The Q-Q plot compares the observed distribution of percent changes in WIC participation with the theoretical normal distribution to assess deviations from normality.

Bivariate and multivariable linear regression analyses were then conducted to examine associations between WIC participation change and state-level factors. The primary model assessed whether states offering WIC delivery services experienced different participation trends compared with those without such programs, controlling for poverty rate, unemployment rate, GDP per capita, demographic composition, educational attainment, urbanization, and political control. An interaction term between delivery availability and poverty rate and unemployment rate was included to test whether the relationship between economic disadvantage and WIC participation changes varied by access to online ordering and delivery services. The distribution of the outcome variable was assessed using histograms, Q-Q plots, and formal normality tests in Stata. The Shapiro–Wilk test (*W* = 0.8735, *p* < 0.001) and the skewness-kurtosis test (χ^2^(2) = 17.87, *p* = 0.0001) indicated deviation from strict normality. Because ordinary least squares regression does not require the dependent variable itself to be normally distributed, models were estimated using OLS with robust standard errors to ensure valid inference under potential heteroskedasticity and distributional deviations (see Figures 4, 5).

Table 3 presents the results of the state-level linear regression models examining changes in WIC participation between November 2024 and September 2025. In the baseline model (Model 1), poverty rate was positively associated with changes in WIC participation (*β* = 0.62, *p* < 0.05), indicating that states with higher poverty levels experienced slightly greater increases in program enrollment. Also, the unemployment rate was negatively associated with changes in WIC participation (β = −1.04, *p* < 0.01), indicating that states with higher unemployment rates experienced significantly smaller increases in program enrollment. The association between delivery availability and participation change was not significant in the base model (model 1).

**Table 3:** Association between food delivery availability and WIC participation rate change.

Variable	Model 1 (no interaction)	Model 2 (delivery × poverty)	Model 3 (delivery × unemployment)
Delivery available (ref: no)	1.71 (2.35)	−105.26 (7.10)***	−22.29 (1.75)***
Poverty rate (%)	0.62 (0.31)*	0.64 (0.31)**	0.64 (0.31)**
Delivery × Poverty rate		11.82 (0.78)***	
Unemployment rate (%)	−1.04 (0.47)**	−1.19 (0.48)**	−1.19 (0.48)**
Delivery × Unemployment rate			6.56 (0.43)***
GDP per capita	−0.00003 (0.00007)	−0.00003 (0.00007)	−0.00003 (0.00007)
Hispanic population (%)	0.11 (0.06)*	0.10 (0.06)	0.10 (0.06)
Black population (%)	0.04 (0.05)	0.03 (0.05)	0.03 (0.05)
White population (%)	0.02 (0.05)	0.01 (0.05)	0.01 (0.05)
Urban population (%)	−0.02 (0.03)	−0.02 (0.03)	−0.02 (0.03)
Republican control (1 = Yes)	−1.09 (0.65)	−1.07 (0.66)	−1.07 (0.66)
Constant	−1.87 (10.10)	−0.38 (10.15)	−0.38 (10.15)
Observations	50	50	50
R-squared	0.421	0.468	0.468

Dependent variable is the percent change in WIC participation from November 2024 to September 2025. Ordinary least squares (OLS) regressions with robust standard errors were adopted. All variance inflation factors are less than 5, indicating no problematic multicollinearity. The Breusch-Pagan test indicated heteroskedasticity (χ^2^ = 9.70, *p* = 0.0018); therefore, robust SEs were applied. *p < 0.05, **p < 0.01, ***p < 0.001.

After adding the interaction term (Model 2), the explanatory power of the model increased (R^2^ = 0.468). The interaction between delivery availability and poverty rate was significant and positive (β = 11.82, *p* < 0.001), suggesting that WIC participation grew more rapidly in high-poverty states that had implemented online ordering and delivery programs. In contrast, the main effect of delivery availability alone was negative (β = −105.26, *p* < 0.001), reflecting that delivery states tended to differ in baseline participation levels. Other covariates, including GDP per capita, educational attainment, urbanization, and political control, were not statistically significant. Additionally, the interaction between delivery availability and unemployment rate was significant and positive (β = 6.56, *p* < 0.001), suggesting that WIC participation grew more rapidly in high-unemployment states that had implemented online ordering and delivery programs.

The negative main effect for delivery availability should be interpreted cautiously. Because the interaction is significant, the main effect simply reflects differences at the reference poverty level rather than an overall negative impact of delivery programs. The model’s improved explanatory power (R^2^ = 0.468) underscores that capturing variation in the poverty and unemployment context is important for understanding where WIC modernization efforts are likely to have the greatest effect. Overall, these findings indicate that WIC modernization through online delivery systems may mitigate participation declines in economically disadvantaged states, highlighting the potential equity benefits of expanding digital access to nutrition assistance.

**Figure 6:**
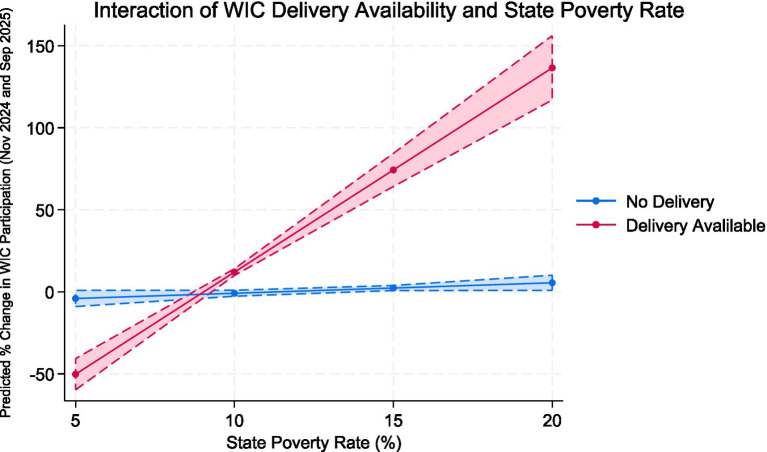
Interaction of WIC delivery availability and state poverty rate on change in WIC participation, 2024–2025. Predicted values are derived from an OLS regression model including controls for unemployment rate, GDP per capita, racial/ethnic composition, college-education rate, and urbanization. Shaded bars represent 95% confidence intervals.

Figure 6 illustrates the interaction between WIC delivery availability and state poverty rate on changes in WIC participation from November 2024 to September 2025. Among states without online ordering or delivery options (blue line), WIC participation remained largely stable across varying poverty levels. In contrast, states with delivery services (red line) exhibited a strong positive gradient: as poverty rates increased, predicted WIC participation rose substantially. At lower poverty levels (~5%), participation was slightly lower in delivery states, but at higher poverty levels (15–20%), predicted participation gains exceeded 75 percentage points compared with non-delivery states. The widening shaded confidence interval reflects greater uncertainty at extreme poverty levels but a consistent overall trend. These results reinforce the regression findings, suggesting that WIC delivery implementation amplifies participation growth in higher-poverty states, potentially mitigating structural barriers to program access among economically disadvantaged populations.

**Figure 7:**
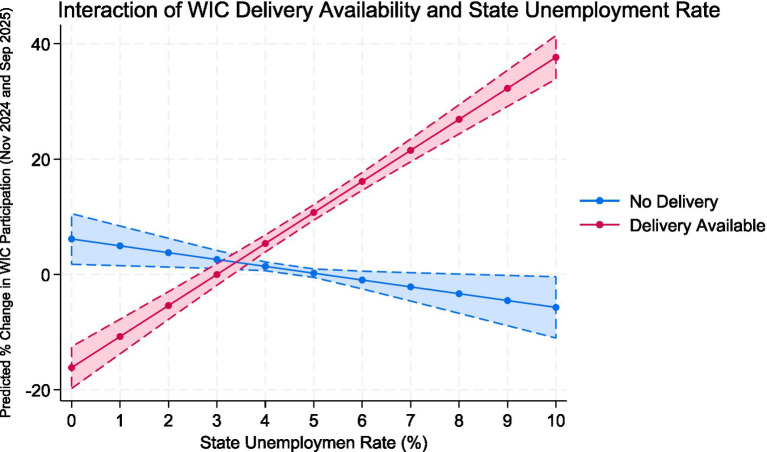
Interaction of WIC delivery availability and state unemployment rate on change in WIC participation, 2024–2025. Predicted values are derived from an OLS regression model including controls for GDP per capita, racial/ethnic composition, college-education rate, and urbanization. Shaded bars represent 95% confidence intervals.

Figure 7 shows how WIC delivery availability and the state unemployment rate interact to influence changes in WIC participation from November 2024 to September 2025. In states without online ordering or delivery (blue line), WIC participation slightly declined across different unemployment levels. Conversely, states with delivery services (red line) demonstrated a strong positive relationship: as unemployment increased, predicted WIC participation rose significantly. At lower unemployment rates (0–3%), participation was marginally lower in delivery states, but at higher unemployment levels (>4%), predicted participation gains surpassed five percentage points compared to non-delivery states. The expanding shaded confidence interval indicates more uncertainty at extreme poverty levels but shows a consistent overall trend. These findings support the regression results, suggesting that implementing WIC delivery boosts participation growth in states with higher unemployment, potentially reducing barriers to program access for economically disadvantaged populations.

## Discussion

WIC participation increased modestly across the United States between 2024 and 2025 but exhibited substantial geographic variation. While several western and mid-Atlantic states experienced sustained growth, other regions saw stagnant or declining participation, reflecting uneven program engagement. Short-term fluctuations suggest that localized implementation efforts and outreach strategies influence participation dynamics. At the same time, persistent regional poverty disparities highlight where barriers to program access remain greatest because high-poverty states are more likely to face structural barriers such as limited transportation and fewer grocery retailers, underscoring the potential value of delivery-based WIC modernization efforts targeted to these regions (12, 35).

This study provides early evidence that implementation of WIC online ordering and delivery systems may help strengthen program participation in socioeconomically disadvantaged contexts. Access to online ordering and delivery was associated with greater gains in WIC participation in higher-poverty states, even after adjusting for socioeconomic and demographic factors. The significant interaction between delivery availability and poverty rate indicates that modernization efforts can help offset structural barriers that depress enrollment and benefit use among families most at risk (29, 36–40).

The result underscores both the short- and long-term benefits of increasing WIC accessibility through digital modernization. Expanded utilization of WIC benefits has been linked to improvements in maternal and child nutrition, reductions in food insecurity, and better long-term health and educational outcomes (5, 10, 41). States with higher poverty rates stand to gain the most from such innovations, as easing logistical barriers, such as transportation and scheduling, can substantially increase program reach among underserved families. Evidence suggests that greater WIC participation contributes to community-level benefits, including improved child health indicators and strengthened local food economies (5, 10, 42).

Importantly, recent technological advances create new opportunities to enhance WIC participation through online benefit redemption and delivery. Massachusetts and Washington have recently piloted online ordering programs in partnership with Walmart, allowing participants to order WIC-approved foods for pickup or delivery across dozens of stores statewide. These early efforts illustrate the feasibility of integrating digital tools into WIC’s infrastructure and provide a model for other states considering similar modernization initiatives. Given the widespread ownership of mobile phones among women of childbearing age, such approaches could meaningfully expand program access, convenience, and retention. Collectively, these findings support the need for future pilot programs and policy efforts aimed at covering delivery or service fees, ensuring that modernization translates into equitable access for families most in need.

The results support three main policy recommendations: (1) extend WIC benefits for use with online ordering and delivery services; (2) subsidize and/or allow WIC benefits to be used to pay for delivery fees, and (3) implement pilot programs guided by geospatial analyses to target communities with families that would most benefit from online ordering and delivery services.

First, extending WIC benefits to support online ordering and home delivery will require user-friendly ordering interfaces, multilingual supports, and training for participants and staff should accompany any rollout to avoid widening a digital divide and ensure equitable digital access (40, 43–45).

Second, delivery fee subsidies or reimbursement could preserve the value of the WIC food package for low-income households, addressing a key cost barrier that programs currently do not uniformly cover (29).

Third, geographically targeted implementation guided by spatial analyses of child population density, transportation difficulty, and retailer participation can prioritize communities where delivery would yield the largest equity gains. In contexts where caregivers face time, mobility, and caregiving constraints, delivery options appear to translate into measurably improved program reach (36, 40). Single mothers with infants, in particular, will be better able to access WIC benefits. For eligible individuals, WIC programs provide food assistance and other services for pregnant, breastfeeding, and postpartum women and their babies and young children.

These findings should be interpreted with caution. The analysis is cross-sectional and ecological, and only a small number of states (two states) had active delivery options during the study period, limiting statistical power and generalizability. We lacked information on household-level eligibility churn, retailer-specific practices (e.g., minimum basket sizes), and whether delivery fees were paid out-of-pocket, factors that plausibly mediate observed effects. Future research should employ longitudinal panel designs and individual-level data to test mechanisms, evaluate fee-subsidy pilots, and examine heterogeneous effects by rurality, race/ethnicity, and family structure (15, 46, 47). Additionally, this policy pilot began in April 2025, meaning the observed period captures early-stage implementation effects rather than long-term program impacts, and state outreach strategies, retailer participation, and implementation practices may influence observed changes in participation. We identify these as important factors for future research.

## Conclusion

The pattern is clear: online ordering and delivery can be effective levers to improve equitable access to WIC, particularly where poverty and transportation barriers are most acute. As WIC modernization accelerates nationwide, policy strategies that subsidize delivery fees, expand retailer participation, enhance digital literacy, and strengthen broadband and device access will be essential to ensuring that these tools benefit all eligible families. Targeting implementation to high-need communities and integrating participant-centered design can help narrow longstanding participation gaps and advance progress toward more equitable child nutrition and health outcomes.

Future research should examine how WIC modernization can be tailored to rural communities, where transportation barriers, sparse retailer networks, limited broadband access, and delivery feasibility may differ substantially from urban settings. Policy-relevant analyses could combine geospatial measures of travel distance, retailer participation, broadband coverage, and delivery service areas with state or county-level participation data to identify communities where delivery options would produce the greatest equity gains. In addition, because the present study models interactions linearly, future work should consider more flexible analytic approaches-such as nonlinear specifications, spatial analyses, and machine learning methods-to better capture complex relationships among poverty, unemployment, geography, and access to WIC delivery services.

## Data Availability

The original contributions presented in the study are included in the article/supplementary material, further inquiries can be directed to the corresponding author.
